# Study of an AC dielectric barrier single micro-discharge filament over a water film

**DOI:** 10.1038/s41598-018-29189-w

**Published:** 2018-07-19

**Authors:** Patrick Vanraes, Anton Nikiforov, Annemie Bogaerts, Christophe Leys

**Affiliations:** 1 PLASMANT, Department of Chemistry, University of Antwerp Campus Drie Eiken, Universiteitsplein 1, 2610 Wilrijk-Antwerp, Belgium; 2 RUPT, Department of Applied Physics, Ghent University, Sint-Pietersnieuwstraat 41 B4, 9000 Ghent, Belgium

## Abstract

In the last decades, AC powered atmospheric dielectric barrier discharges (DBDs) in air with a liquid electrode have been proposed as a promising plasma technology with versatile applicability in medicine, agriculture and water treatment. The fundamental features of the micro-discharge filaments that make up this type of plasma have, however, not been studied yet in sufficient detail. In order to address this need, we investigated a single DBD micro-discharge filament over a water film in a sphere-to-sphere electrode configuration, by means of ICCD imaging and optical emission spectroscopy. When the water film temporarily acts as the cathode, the plasma duration is remarkably long and shows a clear similarity with a resistive barrier discharge, which we attribute to the resistive nature of the water film and the formation of a cathode fall. As another striking difference to DBD with solid electrodes, a constant glow-like plasma is observed at the water surface during the entire duration of the applied voltage cycle, indicating continuous plasma treatment of the liquid. We propose several elementary mechanisms that might underlie the observed unique behavior, based on the specific features of a water electrode.

## Introduction

Dielectric barrier discharges (DBDs) have been intensively investigated for many decades, due to their wide applicability in several industrial processes. Recently, a DBD in contact with a water electrode has received increased attention, since it has been proposed for various promising applications in, among others, water treatment^1–3^, agriculture^4–6^ and medicine^7–9^. As discussed in the review^3^, DBD over a horizontal water electrode is most commonly powered with AC voltage. Moreover, AC high voltage is a popular choice as well for coaxial DBD reactors with falling water film. In the majority of the studies, glass is used as a dielectric barrier, especially quartz glass, and the plasma is operated in air^3,5–7^. A fundamental investigation on this discharge type is therefore interesting, as it can provide better understanding of the process and lead to recommendations for optimized application.

Surprisingly, reports on the diagnostic investigation of DBD plasma with a liquid electrode in atmospheric air are scarce in scientific literature^10^. This discharge type has, however, direct similarities with a DBD in air with solid planar parallel electrodes, such as its usually filamentary nature. This can be concluded, for instance, by comparing the observations for planar parallel DBD over water in^11^ with the features of planar parallel DBD between solid electrodes, as mentioned in^12–14^. For parallel planar or coaxial configuration, DBD plasma consists of numerous filaments, termed micro-discharges. The apparent similarity of DBD over a water surface as compared to DBD between solid electrodes can partly be explained by the dielectric nature of water. Indeed, a water electrode behaves as a dielectric on time scales smaller than the Maxwellian relaxation time *τ* of the solution, which is given by^15^

1 $${\tau }={{\varepsilon }}_{{r}}{{\varepsilon }}_{0}/{\sigma }$$

*ε*_r_ is the relative dielectric constant of water, with a value of *ε*_r_ = 80, ε_0_ is the vacuum permittivity and σ is the solution conductivity. For example, *τ* = 1 µs for a conductivity of *σ* = 7.8 µS/cm and drops to 78 ns for a conductivity of σ = 100 µS/cm. The actual conductivity can be significantly different from the initial conductivity, since plasma treatment of the solution raises the conductivity to a few hundreds of µS/cm. This makes the water electrode act as a dielectric on nanosecond scale, a range in which breakdown occurs for a DBD with solid electrodes. Therefore, the onset mechanism of a DBD over a water surface is expected to be similar to a DBD with a double dielectric barrier.

At atmospheric pressure, the development of a filamentary DBD micro-discharge can be subdivided into three stages^12,14^:

1. During the pre-breakdown stage, negative charge is accumulated in front of the anode until the local electric field strength reaches a critical level. This stage lasts for at least 0.5 µs.
2. Next, an ionization wave propagates towards the cathode. This stage is called the propagation stage and lasts for 1 to 2 ns. As should be noted, cathode-directed positive streamers are characteristic for AC dielectric barrier discharge between solid planar electrodes, independently of voltage polarity during each sine half cycle^12^. Negative streamers, on the other hand, only occur for specific electrode configurations, such as the multipoint-to-plane geometry in^16^.
3. After the discharge filament has bridged the electrode gap, a cathode layer is formed and a bright plasma channel is created. As a result, charge is accumulated on the dielectric surface, compensating the external electric field. This stage is called the decay stage, since it is characterized by the decay of light emission and current pulses.

While the development of a micro-discharge channel occurs in the nanosecond range, plasma chemical reactions by atoms and excited species typically start within the microsecond scale. Production of such plasma species is determined by the reduced local electric field strength *E*/*n* and electron density *n*_e_^14^. Hence, these plasma parameters are essential for understanding and optimizing the overall process.

For a filamentary discharge at atmospheric pressure, the early breakdown stages are similar to those without dielectric barrier. Therefore, the concepts of streamer onset and propagation, as between metal electrodes, are helpful for a better insight. During streamer onset, an electron avalanche can produce substantial charge density after traveling a short distance, because of the high collision rates at atmospheric pressure. For a positive streamer, the propagation mechanisms are generally believed to be a combination of collisional ionization in the region at the streamer head and ultraviolet photoionization of the gas ahead of it^17^. When the streamer is generated in air in strong uniform external fields, photoionization is essential only during the early stage of streamer formation. Collisional multiplication of the generated seed photoelectrons becomes, however, dominant in the later propagation stages^17^. In atmospheric air, the propagating ionizing region at the streamer head has a thickness of 200 µm^18^. In this region, the initial electron density of about 10^12^ cm^−3^ rapidly increases up to the density in the streamer channel of the order of 10^14^ cm^−3^, according to the modeling results in^17^. The reduced field *E*/*n* at the streamer head can reach 500 to 800 Td (with 1 Td = 10^−21^ V m²) and its velocity ranges from 10^7^ to 10^8^ cm/s^13^. When it reaches the opposite electrode, the plasma channel connects both electrodes and becomes more intense. Both the radius of the streamer head and the width of the resulting plasma channel range from 0.1 to 1 mm^13^. At breakdown, the electron density in the filament core remains around 10^14^ to 10^15^ cm^−3^ and the reduced electric field drops to 100–200 Td^12–14^. From that moment on, electron generation at the cathode by photoemission processes is widely substituted with secondary electron emission via positive ion impact. In case of a cathode covered by a dielectric, one should keep in mind the difference between desorption of adsorbed electrons accumulated on the surface and emission of electrons from intrinsic surface levels of the dielectric. Adsorbed electrons have a low binding energy of about 1 eV and a small surface density from 10^10^ to 10^11^ cm^−2^, while intrinsic electrons have a high work function of several eV and a large surface density exceeding 10^16^ cm^−2^ ^19^. As a result of secondary electron emission, cathode layer formation is associated with a current pulse of about 0.1 A. For electronegative gases such as air and oxygen, the shape and amplitude of the current pulse of a single micro-discharge are independent of the applied voltage waveform, as long as the voltage increase rate is slower than about 0.5 kV ns^−1^ ^12^. As a result, the total charge transferred in one micro-discharge filament is in the order of 0.1 to 1 nC and the current density reaches a value of 10^6^ to 10^7^ A m^−2^. While the mean electron energy can reach 1 to 10 eV, the gas temperature of the micro-discharge remains low. Although the local gas temperature elevation inside the micro-discharge channel is theoretically expected to be negligible (Δ*T* = 5 to 10 K)^13,14,20,21^, experimental values up to 230 K are reported in literature. This temperature increase is found to be independent of input power, while it increases with voltage frequency^22^. However, as long as the mean gas temperature is kept low, this temperature rise is sufficiently small for various applications.

Diagnostics of DBD plasma in air are not straightforward, due to its dynamic and inhomogeneous nature. This complicates accurate investigation of the plasma characteristics, such as discharge shape, dimensions, temporal behavior and electron density. To this end, we have developed a DBD reactor where a single micro-discharge filament is generated in between a water film and a quartz glass barrier. A sphere-to-sphere electrode geometry was used in our study, in order to produce a stable DBD micro-discharge filament with fixed location. An additional advantage of such geometry is the possibility to observe not only the filamentary discharge channel, but the surface discharges as well. In this paper, we will discuss the discharge evolution and point out similarities and differences with observations performed by other authors. Spectroscopical analysis is performed to determine the rotational and vibrational temperature *T*_rot_ and *T*_vib_ in the filament, as well as the reduced electrical field strength *E*/*n* and electron mobility *µ*_e_. The temporal and spatial features of the discharge are investigated by means of ICCD imaging, a photomultiplier tube (PMT) and electrical characterization. Finally, after estimation of the current density *j*, we will make an estimation of the electron density *n*_e_ and compare it with other DBD plasmas.

## Results and Discussion

### Temporal discharge properties

**Figure 1:**
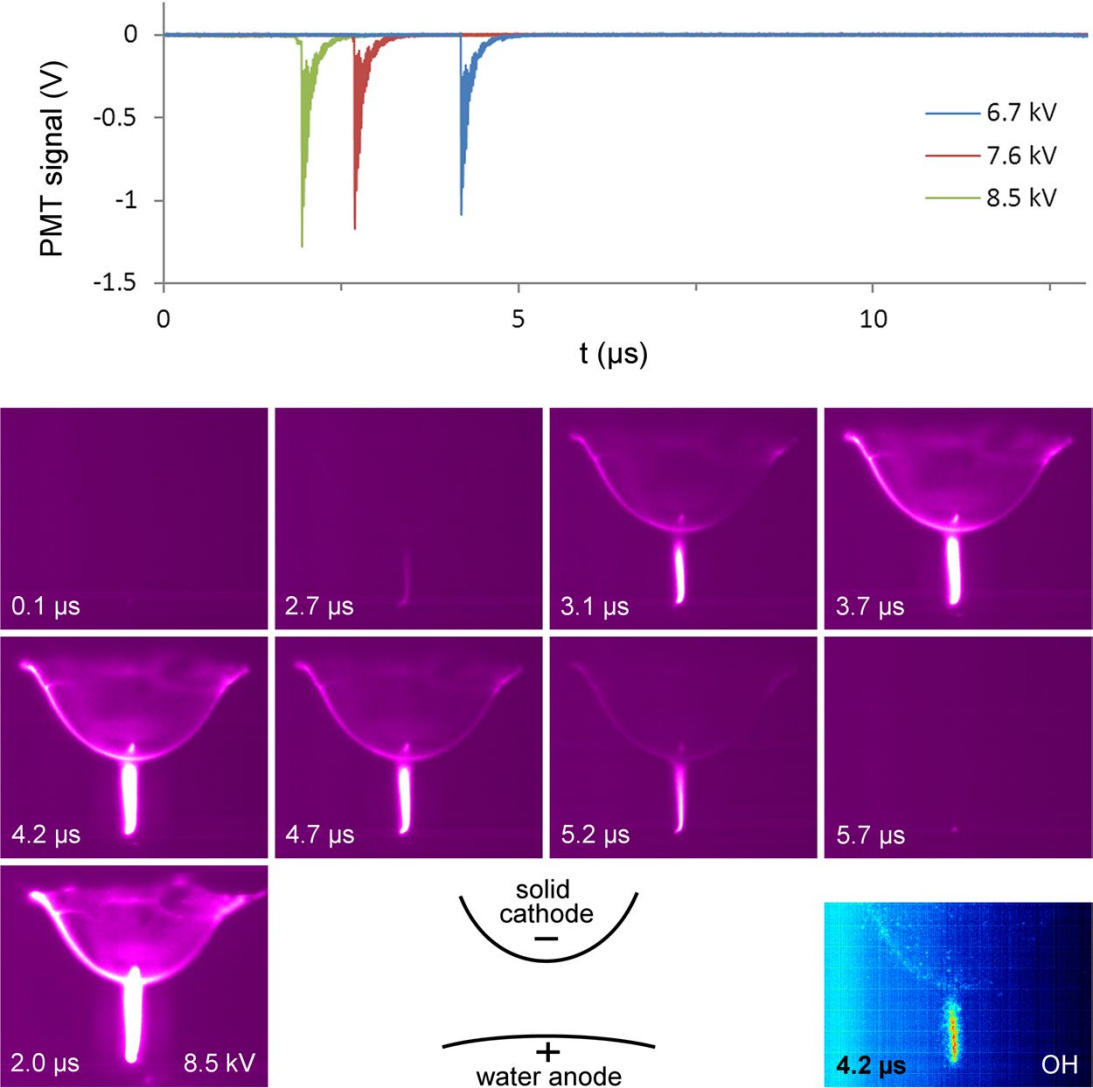
Evolution of plasma light emission during the negative voltage half cycle. (top) PMT output signal at the N_2_(C-B) wavelength of 357.4 nm for different voltages, which are identical in shape to the recordings for the N_2_(C-B) wavelength of 336.9 nm. (bottom) Processed averaged ICCD images with 1.0 µs exposure time, starting at the time indicated in the picture, and a schematic diagram of the setup. The voltage amplitude for all images is 6.7 kV, unless if indicated in the picture (bottom left panel). The plasma evolution as measured for 7.6 kV and 8.5 kV is analogous to the one shown for 6.7 kV, but it appears earlier and brighter with increasing voltage. The snapshot for 8.5 kV is to be compared with the one at 4.2 µs for 6.7 kV. The image with OH filter is found in the bottom right corner. The start of the negative half cycle is chosen as *t* = 0 µs.

Figure 1 shows the discharge evolution during the negative half cycle (i.e. the situation with temporary water anode) for a voltage amplitude of 6.7 kV. Comparison is also made with a voltage amplitude of 8.5 kV in the bottom left panel, and the PMT signals (top panel) are shown for three different voltages. Only one short bright stage of 1.0 µs is seen at the moment of the current pulse. This stage has similar characteristics to a micro-discharge between two solid electrodes, as described in the introduction. After the formation of a conductive channel between the electrodes (t = 2.7 µs in Fig. 1), breakdown occurs and positive charge is deposited on the dielectric surface. This is the only stage where the PMT signal shows emission from the OH(A) band at 309.0 nm (see Figure S1 in the Supplementary information). ICCD images with the filter with central wavelength of 309 nm clearly indicate that OH(A) states are mostly localized in the discharge channel, while dim emission can be seen at the dielectric surface (Fig. 1, bottom right panel).

**Figure 2:**
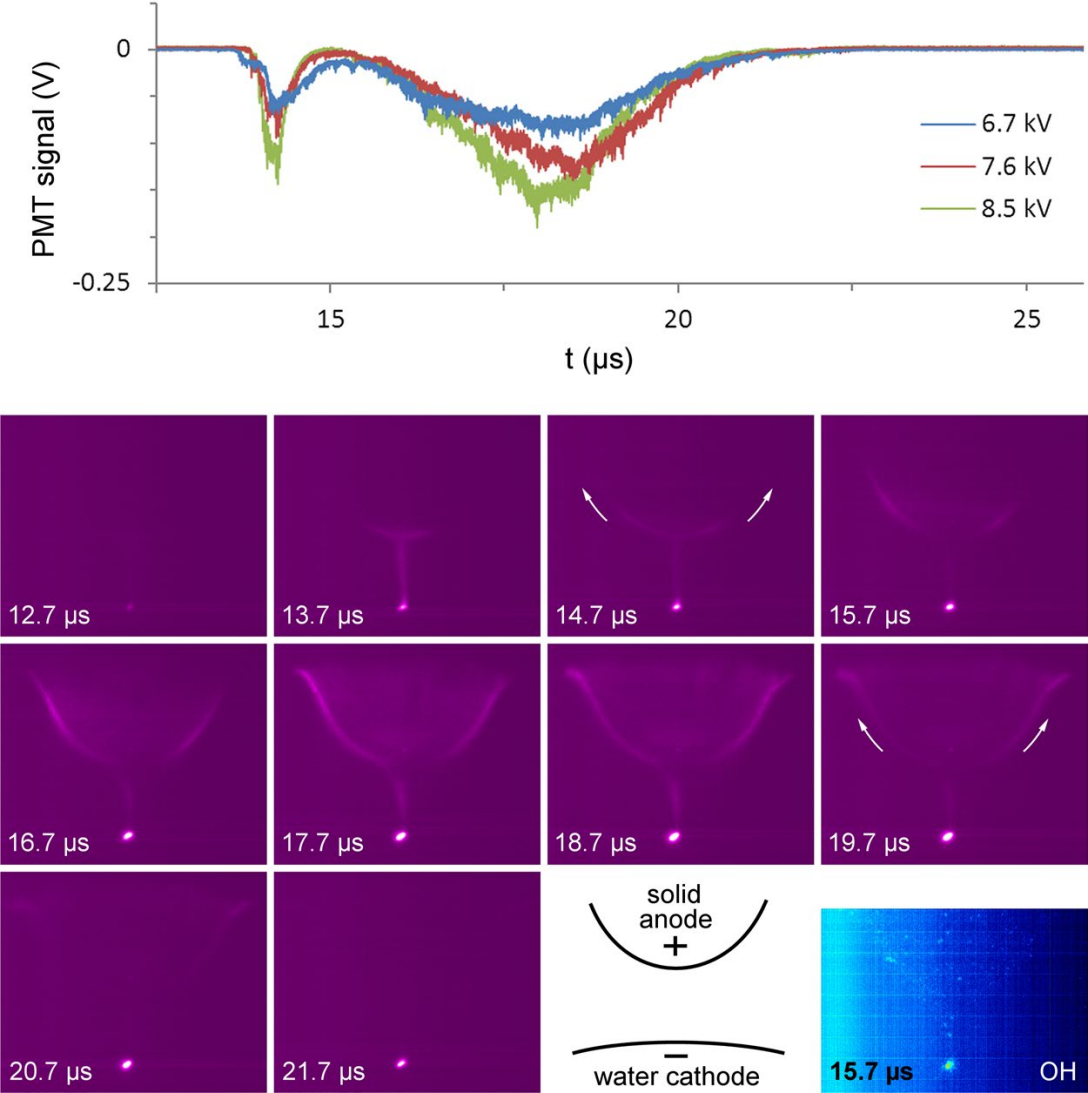
Evolution of plasma light emission just before and during the positive voltage half cycle. (top) PMT output signal at the N_2_(C-B) wavelength of 357.4 nm for different voltages, which are identical in shape to the recordings for the N_2_(C-B) wavelength of 336.9 nm. (bottom) Processed averaged ICCD images for a voltage amplitude of 6.7 kV, starting at the time indicated in the picture, and a schematic diagram of the setup. The plasma evolution as measured for 7.6 kV and 8.5 kV is analogous to the one shown here for 6.7 kV, but it appears brighter with increasing voltage. The exposure time for all images is 1.0 µs, except for the image with OH filter, where it is 5.0 µs. The start of the negative half cycle is chosen as *t* = 0 µs. The positive half cycle starts at 13.6 µs.

During the positive half cycle (i.e. the situation with temporary water cathode), the micro-discharge displays an unusual temporal behavior, as shown in Fig. 2. At a voltage of −0.7 kV, right before the positive half cycle, a short moderately bright stage occurs with duration of 1.3 µs, which is associated with the formation of a plasma channel (t = 13.7 µs in Fig. 2). After the decay of this short bright stage, a long moderately bright stage follows, which manifests itself as a hump with duration of 7.0 µs in the PMT signal, very similar to the photocurrent humps reported in^23^ and^24^. For a voltage amplitude of 8.5 kV, the photocurrent briefly reaches towards zero between both stages, indicating the existence of a short dark stage. At lower voltages, the two bright stages start overlapping and no clear dark stage is measured (see upper panel in Fig. 2). During the decay of the first bright stage, as well as during the entire second bright stage, a glow-like surface discharge propagation is seen, which starts at the upper end of the discharge channel and moves upwards along the dielectric surface. This is indicated with the arrows in the ICCD images and can also be seen in the video in the Supplementary information. At the same time, a cathode spot at the water surface becomes more intense. OH(A) emission is strongly localized in the cathode spot at the water surface (bottom right panel in Fig. 2).

When the amplitude of the applied voltage increases, the bright stages become more intense, while their duration is unaffected. With an amplitude increase from 6.7 kV to 8.5 kV, the onset voltage of the brightest stage during the negative voltage half cycle decreases from −4.3 kV to −2.7 kV (see Figure S2 in the Supplementary information). This change in breakdown voltage is caused by the higher amount of negative charge deposited on the dielectric during the positive half cycle, enhancing the electric field. In contrast, the voltage amplitude has no significant effect on the onset voltage of −0.7 kV of the short bright stage right before the positive half cycle (t = 13.7 µs in Fig. 2). This is a remarkable result, as the low negative voltage at breakdown implies that the critical electric field for breakdown is achieved by positive charge on the dielectric alone, which is deposited during the previous bright stage. This phenomenon is similar to the secondary breakdown during the voltage drop in DBD with square pulses^25^. Nevertheless, it has, to our knowledge, not been reported and discussed before in literature for the case of AC powered DBD. Possibly, this is a phenomenon characteristic for DBD with a water electrode, where special conditions are met, such as a continuous plasma discharge at the water surface.

Remarkably, glow-like light emission is observed on every moment in both voltage half cycles, also during each dark stage, at the location where the micro-discharge channel hits the water surface during breakdown. This indicates that the plasma discharge remains present at all times at the water electrode, although no micro-discharge channel is visible during the dark stages. Within the accuracy of our measurements, OH(A) emission on this location is only observed during the bright stages.

### Peculiarities of the water electrode

Considering the unusual micro-discharge evolution during the positive voltage half cycle, when water acts as a cathode, we hereby postulate five mechanisms that might be responsible for the unique behavior:

1. Local electric field enhancement by water surface deformation;
2. Local electric field enhancement by micro- or nanosized droplets at the water interface;
3. Transfer of aqueous ions to the gas phase by ambient desorption ionization;
4. Stabilization of the cathode fall region by increased local humidity;
5. Stabilization of the cathode fall region by effective secondary electron emission from the water cathode.

We believe that these mechanisms can manifest themselves in the constant glow-like plasma at the water surface, as well as in the glow-like surface discharge propagation associated with the photocurrent hump during the positive voltage half cycle. The former three mechanisms can also explain the continuous plasma at the water surface during the negative voltage half cycle. However, the exact nature of these processes, as well as their occurrence or relative importance, is likely different for opposite voltage polarity. These mechanisms are in agreement with several observations and models reported in literature^19,26–48^. A detailed discussion of the five mechanisms, based on these references, is given in the Supplementary information.

Note that each mechanism appeals to specific features of the water electrode. As a main argument, both discharge phenomena, i.e., the constant glow-like plasma at the water surface and the glow-like surface discharge propagation associated with the photocurrent hump during the positive voltage half cycle, are most likely caused by the unique nature of a liquid electrode, since they have not been observed – or very rarely^23,24^ – in DBD with solid electrodes. They cannot be explained with the electrode geometry, because the electric field is higher at the dielectric barrier than at the water surface according to COMSOL simulation results (see section 2.4) and because they are not observed for similar geometry with solid electrodes^49,50^.

Kozlov *et al*. reported a continuous glow on the solid dielectric surfaces of both electrodes in their reactor during the pre-breakdown stage for more than 500 ns. Nevertheless, this glow discharge was 5 orders of magnitude lower in intensity than the micro-discharge channel^50^. In contrast, the glow discharge in our experiments is only observed at the water electrode and has a similar intensity as the micro-discharge channel. To our knowledge, an intense continuous glow discharge localized at one electrode of a dielectric barrier discharge in air has never been reported in scientific literature for the case of solid electrodes. A photocurrent hump during only one voltage half cycle, on the other hand, has been observed by Miralaï *et al*. and by Li *et al*. in unusual DBD configurations with solid electrodes^23,24^.

In the assumption that electron emission from a water cathode (e.g. due to ion bombardment, photoelectric effect or explosive emission^10^) is a fast process, the mobility of aqueous ions limits the rate at which the loss of negative charge at the water cathode is compensated. As a result, the water cathode is expected to act as a resistive layer for time scales larger than the Maxwellian relaxation time of the solution, as given by Formula 1. This presumably leads to the observed 7.0 µs long hump in the PMT signal. Note that the 7.0 µs corresponds to a quarter of the voltage period and ends at the voltage maximum. This is in good agreement with the observations of other authors. Li *et al*. reported a long hump of several microseconds following a 1 µs pulse in the photocurrent for a filamentary argon plasma in between a metal pin electrode and a resistive water electrode covered with a dielectric plate^23^. The authors explained the hump with the formation of a cathode fall region, which prolongs the discharge life time and leads to the light hump, as in our experiments. Since a cathode fall region is stabilized by a local enhancement of the electric field (see section S1 and S2 in the Supplementary information) or ion density (see section S4), its formation can be favored at one of the electrodes, resulting in a polarity effect. This explains the effect of voltage polarity observed in both^23^ and our experiments. Miralaï *et al*. generated an AC dielectric barrier discharge in pure helium in a plate-to-plate configuration between a quartz barrier and a stainless steel electrode^24^. When the stainless steel electrode was coated with a resistive Al_2_O_3_ + 13% TiO_2_ layer with thickness of 500 µm, similar humps were observed in the photocurrent during both negative and positive voltage half cycles, each following a bright discharge peak. Without the resistive coating, only the peaks occurred, without any hump. We therefore conclude that the occurrence of the photocurrent hump is characteristic for a resistive barrier discharge, while its dependence on voltage polarity indicates that the cathode fall formation is favored at one of the electrodes.

It is important not to misinterpret this polarity effect. Namely, while our results indicate that a water cathode acts as a resistive layer, a water anode seems to behave more like a metal in our experiments. In contrast, Bruggeman *et al*. attributed a resistive character to a water anode, based on stabilization of a DC glow discharge and pattern formation of the anode layer, two phenomena which are observed as well in a resistive barrier discharge with solid electrodes^51^. The difference with our observations on the water resistivity is likely caused by the different electrode configuration and voltage waveform used in the latter report. In the setup of Bruggeman *et al*., a high voltage pin electrode without a dielectric barrier was located over a grounded bulk tap water electrode. The flat water surface in this setup promotes anode layer pattern formation, in contrast to the thin, curved deionized water film in our experiments. Moreover, the duration of the negative voltage half cycle in our experiments is likely too short for anode layer pattern formation, since this phenomenon is characteristic for DC voltage at time scales around 100–500 µs in the case of low water conductivity^51,52^. Therefore, the conditions in our experiments probably prevent the clear observation of the resistive nature of the temporary water anode.

Future research is required to determine the importance and dominance of the mechanisms mentioned above, in the formation of the cathode fall region as well as in the sustainment of the continuous glow discharge. As should be kept in mind, their effect can be significantly different for a water cathode than for a water anode. Apart from the dimmer constant glow discharge at a water anode as compared to at a water cathode in our experiments, such asymmetric behavior can also partly explain the difference in plasma evolution during the negative and positive voltage half cycles. Especially mechanisms that favor field enhancement at a water cathode in comparison to a water anode are interesting from this point of view, as they can be responsible for the low breakdown voltage at the start of the positive half cycle. However, the continuous presence of glow discharge at the water electrode seems to imply a different breakdown mechanism than the one described in the introduction for solid electrodes, regardless of the instantaneous voltage polarity. We namely believe that the bright glow discharge at the water electrode during the dark stages is always accompanied by a very dim glow-like column extended over the electrode gap. Whereas negative charge accumulation in front of the anode during the pre-breakdown stage is provided by a Townsend mechanism in the case of a solid DBD electrode, in our experiments it can be achieved by the glow discharge.

### Temperatures according to spectra fitting

**Figure 3:**
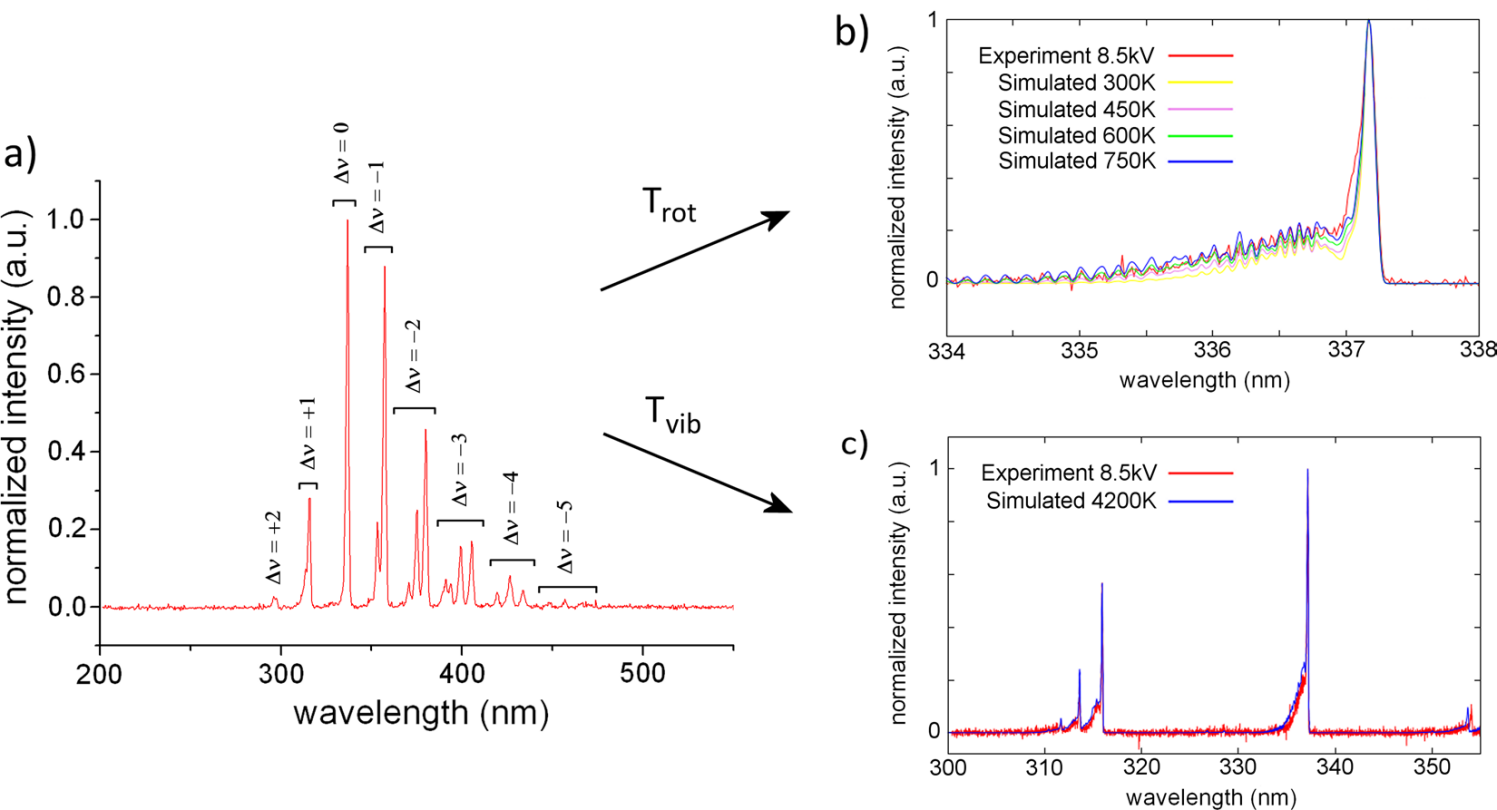
(**a**) Normalized space and time averaged emission spectrum of the micro-discharge for 8.5 kV measured with resolution of 1.3 nm, where Δν represents the change in quantum number ν of each radiative transition in the N_2_(C-B) band system. (**b**) Determination of rotational temperature *T*_rot_ by means of slope fitting of the experimental N_2_ (C-B) band at 338.0 nm. (**c**) Determination of vibrational temperature *T*_vib_ by means of fitting the peak size of the experimental N_2_ (C-B) bands at 315 nm and 338 nm for *T*_rot_ = 750 K.

The rotational and vibrational temperatures *T*_rot_ and *T*_vib_ of the discharge, as obtained from spectra fitting, are summarized in Table 1. The normalized emission spectrum of the discharge, shown in Fig. 3a, is found to be independent of input voltage. The global spectrum is dominated by vibrational bands of the N_2_ second positive band system C ^3^Π_u_(ν′) → B ^3^Π_g_(ν′′). As can be seen from Fig. 3b, the best fitting for 8.5 kV is obtained for a value of *T*_rot_ = 750 ± 150 K. This is in good agreement with the values of 650 K to 850 K obtained in^22^ for a filamentary DBD in a 1 mm electrode gap in air. However, these values were measured for a higher frequency of 60 kHz and the same authors found *T*_rot_ to be dependent on frequency. Dong *et al*. determined rotational temperatures in air atmosphere ranging from 520 to 680 K for an electrode gap from 0.1 to 0.3 mm and voltage amplitude from 4.8 to 6.2 kV at an AC frequency of 40 kHz^53^. In contrast, a value near room temperature was found for a 0.3 mm gap micro-discharge in atmospheric air between cylindrical electrodes with a nanoporous alumina dielectric, driven by 20 kHz AC power at low voltage around 1.7 kV^54^. The reason for this low temperature is the formation of glow plasma instead of filamentary discharge. In^55^, *T*_rot_ is obtained for a pulsed DBD source with interchangeable opposite electrode. When a grounded aluminum plate is used as opposite electrode at 1 mm distance, *T*_rot_ = 400 ± 20 K. On the other hand, *T*_rot_ = 320 K for phosphate-buffered saline solution or glass as opposite electrode. The significantly lower temperature of this discharge is probably due to its pulsed nature and low duty cycle.

**Table 1:** Rotational and vibrational temperature *T*_rot_ and *T*_vib_, reduced electric field *E*/*n* in the middle of the electrode gap, current pulse Δ*I*, mean channel diameter *D* and current density *j* of the micro-discharge filament during the brightest stage, for different values of voltage amplitude *V*.

*V* (kV)	*T*_rot_ (K)	*T*_vib_ (K)	*E*/*n* (Td)	Δ*I* (mA)	*D* (µm)	*j* (10^6^ A m^−2^)
7.5	750 ± 150	4200 ± 500	128 ± 3	60 ± 2	156 ± 10	3.1 ± 0.4
8.5			144 ± 3		173 ± 11	2.6 ± 0.3
9.5			164 ± 3		190 ± 12	2.1 ± 0.3

As shown in Fig. 3c, the best fit is given for *T*_vib_ = 4200 ± 500 K. This is significantly higher than values found in literature from 2600 to 2800 K for 0.1 to 0.3 mm short filaments between glass tube electrodes filled with tap water^53^. As should be noted, the values of *T*_rot_ and *T*_vib_ in our experiments are independent of the applied voltage amplitude in the used range of 7.6 to 9.5 kV, taking into account the accuracy of the measurements and fitting procedures.

### Simulation results

**Figure 4:**
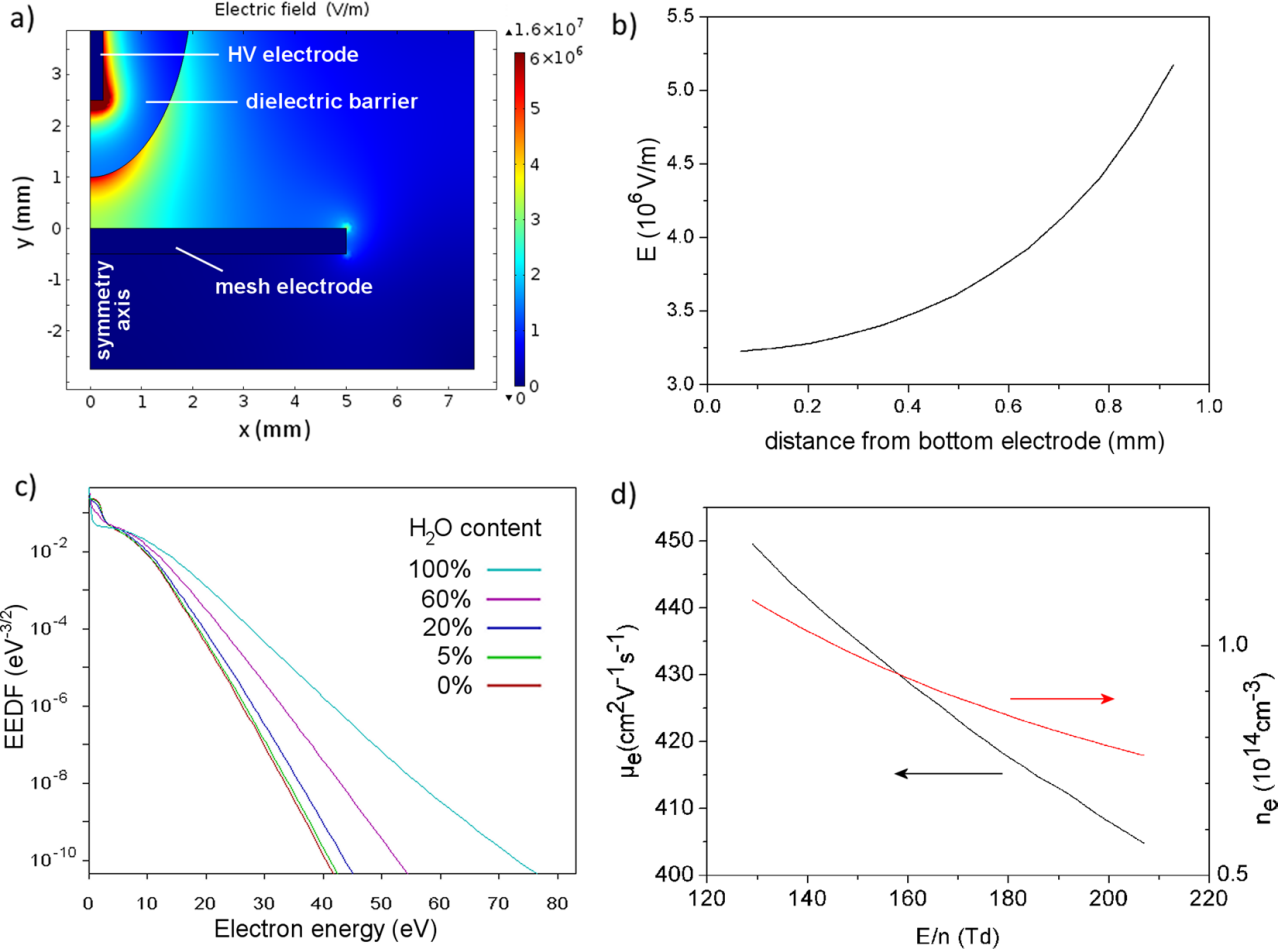
Simulation results from COMSOL and BOLSIG+ for an input voltage of 8.5 kV. (**a**) Electric field distribution in the entire simulation domain. (**b**) Electric field along the symmetry axis of the electrode system. (**c**) Electron energy distribution function (EEDF) determined with BOLSIG+ for air with different contents of humidity, ranging from pure water vapor to dry air, corresponding to the field in the middle of the gap. (**d**) Electron mobility *µ*_e_ and electron density *n*_e_ as a function of the reduced electric field, for *E/n* values found in the interelectrode gap.

The simulation result of the spatial distribution of electric field *E* for 8.5 kV input voltage is shown in Fig. 4a. Figure 4b shows the graph of *E* along the filament core as a function of distance to the grounded mesh electrode (i.e., water electrode in the experiments, but simplified as flat metal for the simulations; see Methods section). Knowing the air number density at atmospheric pressure (*n* = 2.5 × 10^25^ m^−3^), *E*/*n* increases from 132 Td at the mesh electrode to 208 Td at the dielectric barrier covering the pin electrode (i.e., at a distance of 1 mm from the mesh electrode). The results based on *E*/*n* in the middle of the electrode gap for different voltage amplitudes are presented in Table 1; they are in good agreement with the values during breakdown mentioned in literature (100 Td to 200 Td; see introduction). Note that the error on these values is deduced from the experimental error of 2% on the voltage amplitude, used as input in the Comsol simulations (see Methods section). For the pulsed DBD source with interchangeable opposite electrode described in section 2.3, higher values ranging from 300 to 380 Td are found^55^. This higher range is probably due to the higher voltage amplitude of 12 kV. The results for the electron energy distribution function (EEDF) and the electron mobility *µ*_e_ obtained with BOLSIG+, as well as the electron density n_e_, are given in Fig. 4c,d and Table 2. The errors in Table 2 are obtained by taking into account the error on *E*/*n*, and using the latter as input in BOLSIG+ (see Methods section). The EEDF and *µ*_e_ are only slightly dependent on the water content in the gas.

**Table 2:** Product of electron mobility *µ*_e_ and air density *n*, as well as electron density *n*_e_ of the micro-discharge filament for different values of voltage amplitude *V* in dry air, humid air with 5% H_2_O or pure water vapor.

*V* (kV)	gas	*µ*_e_ × *n* (10^24^ V^−1^m^−1^s^−1^)	*n*_e_ (10^14^ cm^−3^)
7.5	dry air	1.119 ± 0.006	1.4 ± 0.2
8.5		1.090 ± 0.005	1.0 ± 0.1
9.5		1.061 ± 0.005	0.8 ± 0.1
7.5	5% H_2_O	1.136 ± 0.006	1.3 ± 0.2
8.5		1.107 ± 0.005	1.0 ± 0.1
9.5		1.075 ± 0.005	0.8 ± 0.1
7.5	pure H_2_O	1.378 ± 0.004	1.1 ± 0.2
8.5		1.356 ± 0.005	0.8 ± 0.1
9.5		1.328 ± 0.004	0.6 ± 0.1

### Current density and electron density

A current peak Δ*I* = 60 ± 2 mA is measured during the most intense stage (see Table 1). This value has no clear dependence on voltage amplitude in our experiments. At this intense stage, a voltage of 8.5 kV yields a channel diameter *D* that increases from 140 µm at the mesh (water) electrode to 200 µm at the dielectric, with an average value of 173 ± 11 µm. This gives a current density of *j* = 2.6 ± 0.3 10^6^ A m^−2^ (see Table 1). As can be seen in Fig. 1, the micro-discharge channel diameter increases with applied voltage. The diameter values in Table 1 are calculated with linear interpolation from the measurements, with *D* = 143 ± 9 µm at 6.7 kV and 173 ± 11 µm at 8.5 kV. The calculated values of *D* and *j*, as given in Table 1, are in good agreement with the range of 0.1 to 1.0 mm and 10^6^ to 10^7^ A m^−2^, respectively, reported in literature as mentioned in the introduction.

As shown in Table 2, the electron density in dry air, humid air and pure water vapor for voltage amplitude of 7.5, 8.5 and 9.5 kV is mostly in the order of *n*_e_ ≈ 10^14^ cm^−3^. This is in good agreement with the values around 10^14^ − 10^15^ cm^−3^ in the filament core of most DBDs with solid electrodes at breakdown (see introduction). For the pulsed DBD source with interchangeable opposite electrode described in section 2.3, a value of *n*_e_ = 2 × 10^15^ cm^−3^ was determined for an interelectrode distance of 1 mm over PBS solution^55^. In comparison, a diffuse atmospheric pressure DC glow discharge with water electrode usually has an electron density around *n*_e_ ≈ 10^13^ cm^−3^, as explained in^56^. Therefore, the values in Table 2 have a realistic order of magnitude, despite the approximations used in the simulations, fittings and averaging for their determination.

## Conclusion

In this study, we investigated an AC powered single DBD micro-discharge filament over a water film by means of electrical, spectroscopical and time- and space-resolved plasma diagnostics in combination with simulations. The filament is generated between a hemispherical quartz barrier and a water layer flowing over a metal grid. Since a water electrode behaves like a dielectric barrier on small time scales in the range of nanoseconds, we compared the breakdown mechanism and properties with those of a filamentary DBD micro-discharge with solid electrodes.

The discharge evolution during the positive voltage half cycle is remarkably different from the one usually observed for DBD in between solid electrodes. While in the negative voltage half cycle, one intense light pulse is seen, as in the solid electrode case, the positive half cycle starts with a less intense light pulse followed by a hump in the photocurrent. The hump is associated with an intense cathode spot at the water surface and a glow-like surface discharge propagation that moves upwards along the dielectric surface, indicating gradual charge deposition on the barrier. Based on two other scientific reports of similar photocurrent humps in AC powered DBD, we can conclude that the phenomenon is characteristic for a resistive barrier discharge, where one electrode acts as a resistive barrier. In our experiments, the water electrode behaves as a resistive layer, an observation which has been reported as well by Bruggeman *et al*.^51^. As cathode fall formation can be favored at one electrode in asymmetric electrode configurations, the photocurrent hump is for a certain voltage range present in only one voltage half cycle, while absent in the other. In our experiments, cathode fall formation only occurs at the water cathode during the positive voltage half cycle. This preference can have many underlying reasons, including local field enhancement by water surface deformation or droplet formation, local ionization enhancement by the inhomogeneous humidity profile and more effective secondary electron emission at the water electrode than at the dielectric barrier. Similar mechanisms might also explain the glow-like discharge spot which is continuously observed at the water electrode during the entire voltage cycle.

For the micro-discharge filament under study, optical emission spectroscopy gives a rotational and vibrational temperature of *T*_rot_ = 750 ± 150 K and *T*_vib_ = 4200 ± 500 K for the second positive N_2_ (C-B) system. By means of simulation with COMSOL, the reduced electric field *E*/*n* in the middle of the electrode gap is calculated to increase from 128 to 164 Td with voltage amplitude from 7.5 to 9.5 kV. The electron mobility *µ*_e_ is determined with BOLSIG+ and the current density *j* is calculated from the current pulse and micro-discharge channel diameter during the brightest discharge stage. An electron density in the order of *n*_e_ ≈ 10^14^ cm^−3^ is deduced from the obtained values of *j*, *µ*_e_ and *E*. All these results are in good agreement with values reported in scientific literature on DBD microfilaments in atmospheric air between solid electrodes.

This study of a single DBD micro-discharge filament helps to gain better insight in the behavior of a DBD with water electrode, which is gaining increasing interest for various applications, such as water treatment, agriculture and medicine.

## Methods

### Experimental arrangement and diagnostic methods

**Figure 5:**
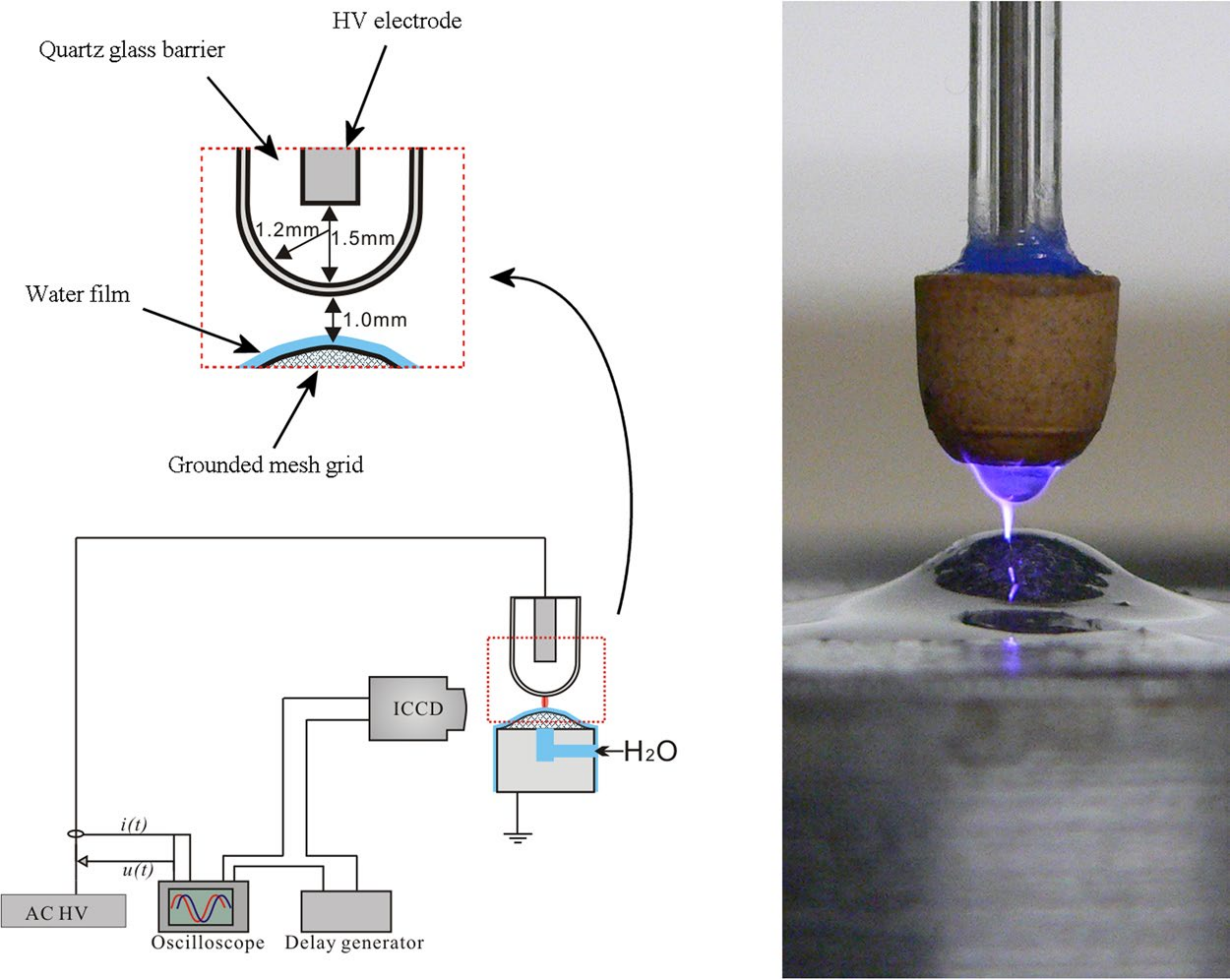
(left) Reactor schematic with dimensions, drawn by Dr. Xiaolong Deng (Changsha, China). The ICCD camera also indicates the position of the optical fiber leading towards the PMT. (right) Photo of the electrode system with discharge.

The setup and its dimensions are shown in Fig. 5. The upper electrode consists of a 0.75 mm thick sharpened high voltage pin electrode in a quartz tube. The downside of the tube is closed and has a spherical shape with a 1.2 mm radius, which acts as dielectric barrier. It is positioned over a grounded spherical metal mesh. Deionized water is slowly pumped upwards through the mesh, covering the electrode with a thin water layer. The interelectrode distance is kept 1.0 mm, unless mentioned otherwise. The micro-discharge filament is generated in surrounding air atmosphere by applying an AC high voltage with amplitude of 6.7 kV, 7.6 kV or 8.5 kV and frequency of 36.9 kHz on the upper pin electrode by means of a custom-made power source. Voltage waveforms are measured with a Tektronix P6015 HV probe and current waveforms with a Pearson model 2877 current probe. Both probes are connected to a Tektronix S1200 oscilloscope. The output signals of the ICCD camera and the PMT are monitored with the same oscilloscope. Optical emission spectra are obtained by means of an Ocean Optics spectrometer S1000 in the range of 250 nm to 900 nm with instrumental full-width at half maximum of 1.3 nm. For higher resolution in the range of 300.0 nm to 355.0 nm, spectra are measured with an Avantes spectrometer (AvaSpec-3048) with instrumental full-width at half maximum of 0.05 nm. ICCD imaging is performed with a Hamamatsu ICCD camera (model C8484) connected to a delay generator, for which the voltage waveform was used as trigger signal. To investigate the space-resolved evolution of light emission of the excited OH(A) states, a filter with central wavelength of 309 nm and bandwidth of 10 nm is fixed in front of the camera. The ICCD images are averaged over 100 accumulations without filter and over 325 accumulations with OH filter. Space-averaged time-resolved light emission at specific wavelengths of the OH(A) band (309.0 nm) and two N_2_ (C-B) bands (336.9 and 357.4 nm) is obtained by means of a photomultiplier tube (PMT) with response time of 50 ns connected to a monochromator. The bandwidth of the monochromator is determined with a low pressure Hg spectral calibration lamp to be 1.5 nm.

### Spectra fitting

The high-resolution spectra obtained with the Avantes spectrometer are processed with the custom-made synthesis software for N_2_ (C-B) transition spectra simulation in order to obtain rotational and vibrational temperature of the filament. The rotational temperature *T*_rot_ can be considered to be equal to the gas temperature inside the micro-discharge channel. It is found by fitting the experimental slope of the N_2_ (C-B) band at 338.0 nm to the simulated one. Subsequently, *T*_vib_ is determined by fitting the amplitude of the N_2_ bands for the resulting value of *T*_rot_ = 750 K.

### Simulation by means of Comsol

The electric field E and charge density in a vertical plane of symmetry through the electrode system are calculated with a fluid model, using the simulation software Comsol Multiphysics (version 4.0). This fluid model is based on conservation equations for mass density, momentum and energy, coupled with Poisson’s equation. The electrode configuration is simulated by simplifying the grounded mesh electrode with water layer as a flat solid metal. This is supposed to give a limited error in the results, due to the small dimensions of the water layer and due to the large curvature of the mesh electrode in comparison to the micro-discharge radius and the dielectric barrier curvature. The metal parts of the electrode system are simulated with the standard material *Armco Iron* from the material library of Comsol. In order to study the electric field *E* on the symmetry axis in the electrode gap, a vertical cutline is applied in the simulation. The reduced electric field *E*/*n* is calculated using the air density *n* = 2.5 × 10^25^ m^−3^ at standard conditions. For further calculations, the value of *E*/*n* in the middle of the electrode gap is used. The error on this value is deduced from the experimental error of 2% on the voltage amplitude, by entering the voltage values ±2% as input of the simulation with Comsol.

### Calculation of EEDF and µ_e_ by means of BOLSIG+

The electron energy distribution function (EEDF) and electron mobility *µ*_e_ are determined by means of the BOLSIG + electron Boltzmann equation solver^57^ (version 06/2013), using the cross section data sets from Morgan (Kinema Research Software)^58^ for N_2_ and H_2_O and from the IST-Lisbon database^59^ for O_2_. The required input parameters are the above values of *T*_rot_ and *E*/*n*, as well as the gas composition. For the gas composition, several mixtures of dry air (78.8% N_2_ and 21.2% O_2_) with variable water vapor content are distinguished. The error on the electron mobility is calculated by taking into account the error on *E*/*n* and using the corresponding values ± error as input in BOLSIG+.

### Determination of filament diameter D and current density

The current density *j* through the micro-discharge filament is calculated from the filament diameter and the current peak Δ*I* during the most intense stage, i.e. the difference between the current with plasma and the displacement current without plasma. Namely, Δ*I* represents the current through the filamentary channel at the corresponding discharge stage. The diameter *D* of the channel at the corresponding discharge stage is deduced from ICCD images.

### Calculation of electron density

The electron density during the most intense micro-discharge stage is determined from the current density *j*, electric field *E* and electron mobility *µ*_e_ as

2 $${{n}}_{{e}}=\frac{{\rm{j}}}{{\rm{E}}\times {{\mu }}_{{e}}\times {e}}$$

### Data Availability

The datasets generated during and/or analyzed during the current study are available from the corresponding author on reasonable request.

## Electronic supplementary material

Supplementary information

video.mp4
