# Supplementary material for: Study of an AC dielectric barrier single micro-discharge filament over a water film
Source: Sci Rep. 2018 Jul 19;8:10919. doi: 10.1038/s41598-018-29189-w (PMC6053385; doi:10.1038/s41598-018-29189-w)
Supplement: Supplementary file 1 — Supplementary information [file 41598_2018_29189_MOESM1_ESM.pdf]

# Study of an AC dielectric barrier single micro-discharge filament over a water film

Patrick Vanraes[1,2,*], Anton Nikiforov[2], Annemie Bogaerts[1], Christophe Leys[2]

[1] *PLASMANT, Department of Chemistry, University of Antwerp Campus Drie Eiken, Universiteitsplein 1, 2610 Wilrijk-Antwerp, Belgium*

[2] *RUPT, Department of Applied Physics, Ghent University, Sint-Pietersnieuwstraat 41 B4, 9000 Ghent, Belgium*

* Corresponding author. tel: +3232652364; fax: +3232652343; email: patrick.vanraes@uantwerpen.be; address: PLASMANT, Department of Chemistry, University of Antwerp Campus Drie Eiken, Universiteitsplein 1, 2610 Wilrijk-Antwerp, Belgium

E-mail addresses: Patrick.Vanraes@uantwerpen.be (P. Vanraes); Anton.Nikiforov@ugent.be (A. Nikiforov); Annemie.Bogaerts@uantwerpen.be (A. Bogaerts), Christophe.Leys@ugent.be (C. Leys).

## Supplementary information

A. Supplementary figures

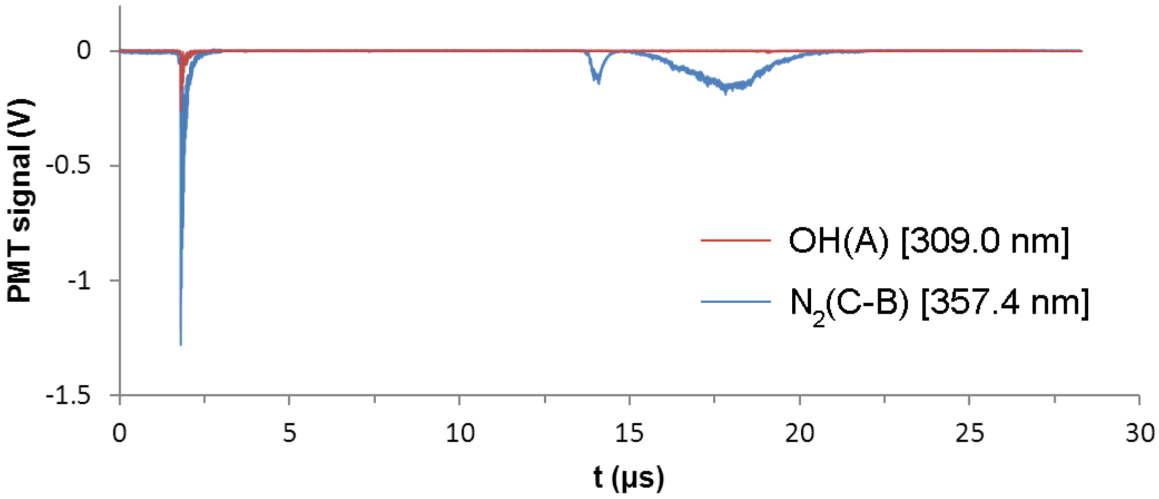


**Figure S1** Space-averaged time-resolved light emission from the micro-discharge filament during one voltage sine cycle with amplitude of 8.5 kV, as measured by means of the PMT at the wavelengths 309.0 nm (corresponding to the OH(A) band) and 357.4 nm (corresponding to a $N_2$(C-B) band). The start of the negative half cycle is chosen as $t$ = 0 µs.

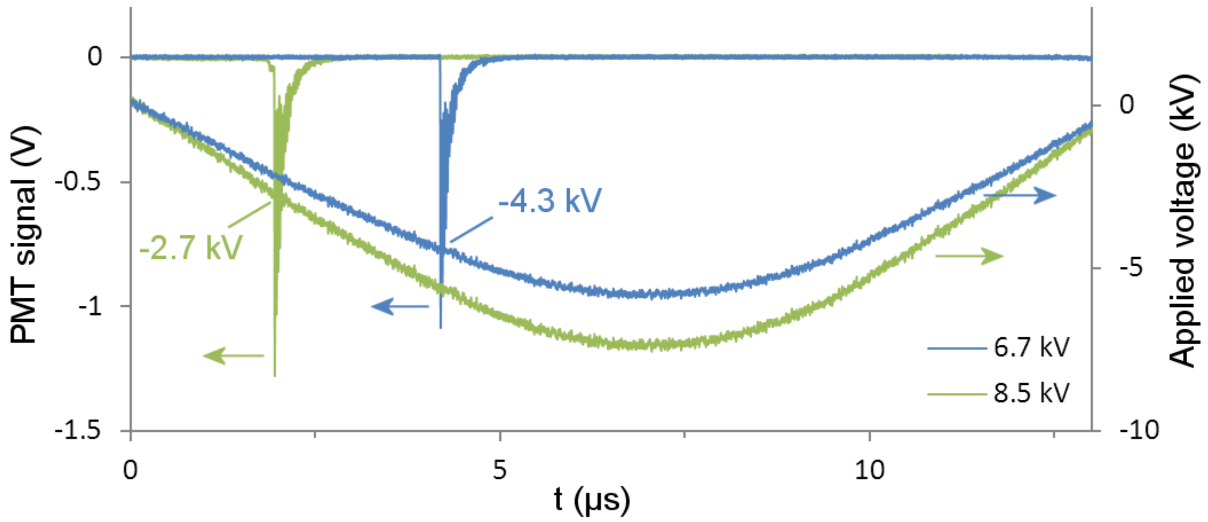


**Figure S2** PMT signal at 357.4 nm and the corresponding applied voltage during the negative voltage half cycle, for the two applied voltage amplitudes 6.7 and 8.5 kV. For both voltage amplitudes, the onset voltage of the short intense stage is given at the intersection of the curves. The start of the negative half cycle is chosen as $t$ = 0 µs.

B. Supplementary video

See the attached file video.mp4. It first shows the negative and then the positive voltage half cycle.

C. Discussion of the five mechanisms that might be responsible for the unusual micro-discharge evolution during the positive voltage half cycle of the single DBD micro-discharge filament, when water acts as cathode.

## S1 Local electric field enhancement by water surface deformation

Surface deformation of a water electrode can result from a combination of local disturbances, such as strong attraction of the local electric field, the impact of accelerated plasma species and the elevated gas temperature during breakdown to several hundreds of degrees above the boiling point of water. Such deformations do not only allow a decrease in interelectrode distance, they can also significantly enhance the local electric field by the formation of sharp electrode points with small radius of curvature. For example, Lu and Laroussi observed the formation of ripples on a water cathode under influence of a glow-like discharge due to the high voltage drop over the cathode fall region [1]. They suggested that the ripples offer sharp curvature points with high electric fields, which can initiate several discharges. Interestingly, ripples did not occur when water acted as anode. However, the formation of these ripples is relatively slow, as it was observed at larger timescales (100 µs and higher). In our experiments, on the other hand, the stable glow discharge in the course of 1 to 10 µs can only be explained with surface deformation if this deformation has a relatively static nature and can be reproduced in every voltage cycle. This could be the case for a static Taylor cone [2]. The typical timescale for Taylor cone formation on a mercury surface is 0.85 µs according to the model of Suvorov [3]. Experimental investigation of Taylor cone formation on a water drop and on water surfaces shows that the instability develops on a timescale of typically 1 to 10 µs [4,5]. The exact characteristic formation time is suggested to depend on electric stress, initial surface shape, and liquid properties such as surface tension, viscosity, density and conductivity [6]. However, these data apply to Taylor cones under a DC electric field, while an AC field with frequency $f > 10$ kHz can generate stable cones with a much sharper top angle [2]. Further investigation is required to determine whether Taylor cone formation plays an important role in our setup and in a DBD with water electrode in general, since it was not observed within the accuracy of our measurements. As an additional advantage of a liquid electrode, a sharp surface deformation can maintain its curvature and surface properties over long periods in time, in contrast to solid electrodes that can be eroded and oxidized. This can also explain why a similar glow-like regime is not observed in DBD with solid electrodes.

## S2 Local electric field enhancement by micro- or nanosized droplets

Similarly to electrode surface deformation, micro- or nanosized droplets can be created at the water interface under influence of an external electric field and plasma processes. Since the local electric field is significantly enhanced at a droplet interface, a lower external field is required for ionization of the surrounding gas and acceleration of charged species up to dissociative energy levels. Such plasma-induced droplet formation is in agreement with some observations on an electrolyte cathode atmospheric glow discharge, which is mostly investigated as a promising technique for monitoring toxic heavy metal content of water. Cserfalvi, Mezei and co-workers have proposed a model where metal vapor is introduced into the gas phase as a consequence of water bombardment by positive gas ions and metastable atoms coming from the cathode dark space and the negative glow [7,8]. Yagov et al., on the other hand, proposed an electrothermal sputtering model where the introduction of small aerosol particles into the plasma is the limiting step in the analytical signal. Their model is based on the observation that the analytical signal is enhanced with increasing temperature and decreasing flow rate of the solution, which both improve thermal sputtering [9]. Nevertheless, these models have been disputed by other authors, such as Marcus and Davis, who cited volatilization of the analyte species due to Joule heating caused by current flow across the liquid–plasma interface as the key mechanism

[10,11]. Either way, sputtering of a water anode has never been suggested in literature and is expected to be less likely, as the electric field of a glow discharge near the anode is significantly lower than at the cathode. Although we agree that anode sputtering is less likely than cathode sputtering, we do not exclude its possibility. Water vapor is namely an electronegative gas that has the characteristic ability to form stable negative cluster ions in the discharge gap [12,13]. When these negative ions are accelerated and reach the anode surface with sufficient energy, sputtering can occur. Yet, the magnitude of sputtering at a water anode through this mechanism is expected to be lower as compared to at a water cathode, due to the relatively low density of negative ions as compared to positive ions. This can explain why the continuous glow discharge in our measurements is dimmer during the long dark stages when water is acting as anode, in comparison to the bright stages and short dark stage of the positive half cycle, when water is the cathode.

Next to this, we hereby propose a novel, alternative sputtering mechanism that might occur specifically for AC powered plasma at a water electrode, as in our experiments. According to this mechanism, negative micro- or nanodroplets are initially formed at a water cathode during the positive voltage half cycle, due to incident positive ions. The negative droplets that survive until the negative voltage half cycle will subsequently be accelerated towards the temporary water anode. Bombardment of the water surface by these droplets will result in sputtering of the water anode, yielding positive droplets. The positive droplets that survive until the positive voltage half cycle will, on their turn, be accelerated back towards the water cathode, generating negative droplets, in addition to the ones produced from positive ions. The size and/or number of the produced droplets are expected to grow as a function of the size and/or number of the incident projectiles. As such, the sputtering magnitude should increase during the first cycles until a dynamic equilibrium has been reached in the droplet size and number.

**S3 Ambient desorption ionization**

Local enhancement of the electric field at the water surface during the dark stages can lead to so-called ambient desorption ionization, where ions are transferred from the liquid to the gas phase by means of highly charged droplet ejection. To understand the underlying mechanism, insights can be taken from recent fundamental research on electrospray ionization, as in [14]. Here, intermediate droplets are dispersed into the gas phase as an aerosol under influence of Coulomb repulsion. Subsequently, they decrease in size through a combination of evaporation and fission processes. The final production of gas-phase ions has been explained with several competing models. In the charge residue model, a droplet decreases in size through evaporation until it reaches its Rayleigh limit, where it undergoes Coulomb fission. After the droplet has diverged into smaller droplets, the cycle continues until a charged solute residue is formed. In the ion evaporation model, on the other hand, direct desorption of solvated ions from the droplet is assumed when the field strength at the droplet surface reaches a threshold value for ion field evaporation. From theory and experiment, this value is calculated to be about 1 V/nm, which is 3 orders of magnitude higher than the field strength at the water interface according to our COMSOL simulation. Such high field strength is therefore expected only at the surface of droplets with a diameter smaller than 10 nm [14]. Electric field induced ion evaporation has been demonstrated for dielectric liquids in [15]. As molecular dynamics simulations have shown, ion field evaporation is also strongly dependent on temperature, in the range of 350 to 800 K [16]. A growing body of evidence suggests that ion field evaporation is mostly applicable for small ions, as in our experiments, while larger ions are more likely formed according to the charge residue model.

## S4 Cathode stabilization by increased local humidity

Under influence of the elevated temperature at the micro-discharge core during the breakdown stages and of the continuous glow during the dark stages, evaporation increases the local water vapor concentration at the water interface. Minor concentration variations or density fluctuations can lead to significant differences in plasma processes. In particular, humidity can strongly influence the formation of a cathode fall region, which is the most important criterion for a glow discharge. If we assume that the glow-like discharge wave during the photocurrent hump can be modeled as a normal glow discharge, positive ions generated at the instantaneous anode travel towards the instantaneous cathode and collide with the cathode to produce secondary electrons. With the arrival of the ions, the net electric field is influenced by these space charges and most of the voltage is located over the cathode fall region, i.e. the gap between the cathode and the space charge layer. In the cathode fall region, ions can gain sufficient kinetic energy through acceleration to produce secondary electrons, while electrons can cause an electron avalanche even at relatively low applied voltage. In this way, the voltage required to maintain the discharge is significantly reduced. Consequently, the glow discharge life time is prolonged until the voltage over the cathode fall region drops under a critical value. This manifests itself in the occurrence of the light hump. The higher the ion density at the cathode, the easier it is for the cathode fall region to form. When the dielectric barrier acts as cathode, accumulation of surface charges can prevent the formation of a cathode fall region, with fast quenching of the discharge. At a water cathode, however, such charge accumulation is not expected.

In a normal glow discharge, the cathode fall length d can be expressed as [17]

$$d = \frac{1}{\alpha}\ln\left(1 + \frac{1}{\gamma}\right) \tag{S4.1}$$

where α represents the first Townsend coefficient and γ is the secondary electron emission coefficient. A higher value of α leads to a smaller cathode fall region, which is beneficial for the stability of the glow discharge. For air ionization, α increases with relative humidity, as explained in more detail in [18]. Since the local relative humidity is higher at the water surface than at the dielectric barrier, so is α. One should, however, keep in mind that electron attachment to electronegative gas molecules in the cathode fall region counteracts this effect, analogously to a decrease of γ in Formula S4.1 [19]. That is, Formula S4.1 is, strictly speaking, only valid for electropositive gases. Nonetheless, the counter-effect by electron attachment only has a small contribution to the change in cathode fall length. It is namely an established experimental fact that the cathode fall length decreases when an electronegative gas (such as $O_2$ and $H_2O$) is added to an electropositive gas (such as $N_2$), on the condition that the former is easier to ionize than the latter [19]. This is indeed the case for $O_2$ and $H_2O$, which both have lower ionization energy than $N_2$ [18]. Accordingly, cathode fall region formation is favored at the water electrode in our experiments due to the inhomogeneous humidity profile.

## S5 Cathode stabilization by effective secondary electron emission from the water cathode

Despite the above mentioned mechanisms, the low breakdown voltage at the start of the positive half cycle is possibly due to energy efficient secondary electron emission at the water cathode. Although the breakdown occurs at a slightly negative voltage of -0.7 kV, the water electrode acts as a cathode because of the positive surface charge deposited on the dielectric during the negative half cycle. In case of a water cathode, secondary electron emission occurs through an essentially different mechanism than in the case of solid electrodes. The quartz barrier has a work function around 5 eV [20,21], which possibly requires a significantly higher voltage for breakdown. As a side-note, desorption of accumulated electrons on the dielectric surface takes less energy, around 1 eV, but this process is probably too limited before the moment of breakdown due to the lack of initiating mechanisms such as

interactions with vibrationally excited $N_2$ and thermodesorption [22]. Because of the low surface density of adsorbed electrons, such desorption processes are more likely during the following bright micro-discharge stage, when the excited $N_2$ density and local temperature of the dielectric increase.

From Formula S4.1, it can easily be seen that a higher value of $\gamma$ leads to a shorter cathode fall region, which is beneficial for the stability of the glow discharge. This qualitative trend is expected to be valid for both electropositive as electronegative gases, since the value of $\gamma$ depends on the cathode material. In [23], $\gamma$ is estimated to be 0.01 for a quartz glass dielectric barrier, based on theory and simulation results. For a water cathode, on the other hand, the apparent value of $\gamma$ depends on pH. According to [8,24], $\gamma$ decreases from 0.18 at pH = 0.3, over 0.07 at pH = 1.71, to a constant value $\gamma = 0.02$ for pH = 2.5 or higher. In our system, the local pH at the glow discharge can be very low, as DBD plasma discharge in air is known to increase the overall solution acidity to values below pH = 3, but an exact value for $\gamma$ is unknown. In any case, these calculations suggest a shorter and more stable cathode fall region at a water cathode than at the dielectric barrier.

**References**


1 Lu, X. & Laroussi, M. Ignition phase and steady-state structures of a non-thermal air plasma. *Journal of Physics D: Applied Physics* **36**, 661 (2003).

2 Demekhin, E., Polyanskikh, S. & Ramos, A. Taylor cones in a leaky dielectric liquid under an ac electric field. *Physical Review E* **84**, 035301 (2011).

3 Suvorov, V. & Litvinov, E. Dynamic Taylor cone formation on liquid metal surface: numerical modelling. *Journal of Physics D: Applied Physics* **33**, 1245 (2000).

4 Bruggeman, P. *et al.* DC electrical breakdown in a metal pin–water electrode system. *Plasma Science, IEEE Transactions on* **36**, 1138-1139 (2008).

5 Giglio, E. *et al.* Shape deformations of surface-charged microdroplets. *Physical Review E* **77**, doi:10.1103/PhysRevE.77.036319 (2008).

6 Kim, J., Oh, H. & Kim, S. S. Electrohydrodynamic drop-on-demand patterning in pulsed cone-jet mode at various frequencies. *Journal of Aerosol Science* **39**, 819-825, doi:10.1016/j.jaerosci.2008.05.001 (2008).

7 Cserfalvi, T., Mezei, P. & Apai, P. Emission studies on a glow discharge in atmospheric pressure air using water as a cathode. *Journal of Physics D: Applied Physics* **26**, 2184 (1993).

8 Mezei, P. & Cserfalvi, T. Electrolyte Cathode Atmospheric Glow Discharges for Direct Solution Analysis. *Applied Spectroscopy Reviews* **42**, 573-604, doi:10.1080/05704920701624451 (2007).

9 Yagov, V., Getsina, M. & Zuev, B. Use of electrolyte jet cathode glow discharges as sources of emission spectra for atomic emission detectors in flow-injection analysis. *Journal of Analytical Chemistry* **59**, 1037-1041 (2004).

10 Davis, W. C. & Marcus, R. K. An atmospheric pressure glow discharge optical emission source for the direct sampling of liquid media. *Journal of Analytical Atomic Spectrometry* **16**, 931-937, doi:10.1039/b103437p (2001).

11 Marcus, R. K. & Davis, W. C. An atmospheric pressure glow discharge optical emission source for the direct sampling of liquid media. *Analytical Chemistry* **73**, 2903-2910 (2001).

12 Gallimberti, I. The mechanism of the long spark formation. *Le Journal de Physique Colloques* **40**, C7-193-C197-250, doi:10.1051/jphyscol:19797440 (1979).

13 Bruggeman, P., Iza, F., Lauwers, D. & Gonzalvo, Y. A. Mass spectrometry study of positive and negative ions in a capacitively coupled atmospheric pressure RF excited glow discharge in He–water mixtures. *Journal of Physics D: Applied Physics* **43**, 012003 (2009).

14 Luedtke, W. *et al.* Nanojets, electrospray, and ion field evaporation: Molecular dynamics simulations and laboratory experiments. *The Journal of Physical Chemistry A* **112**, 9628-9649 (2008).

15 Gamero-Castaño, M. Electric-Field-Induced Ion Evaporation from Dielectric Liquid. *Physical Review Letters* **89**, doi:10.1103/PhysRevLett.89.147602 (2002).

16 Longhi, G. *et al.* Molecular dynamics of electrosprayed water nanodroplets containing sodium bis(2-ethylhexyl)sulfosuccinate. *Journal of Mass Spectrometry* **48**, 478-486, doi:10.1002/jms.3179 (2013).
17 Schoenbach, K. H. in *Low temperature plasma technology: methods and applications* (eds Paul K Chu & XinPei Lu) (CRC Press, 2013).
18 Dogu, I. Fundamentals of Electrostatic Spinning Part IV:-Ionization of Atmospheric Air under Action of an Electric Field. *Textile Research Journal* **54**, 111-119 (1984).
19 Duke, G. L. Investigation of Sheath Phenomena in Electronegative Glow Discharges. (AIR FORCE WRIGHT AERONAUTICAL LABS WRIGHT-PATTERSON AFB OH, 1985).
20 Quinn, J. W., Captain, J. G., Weis, K., Santiago-Maldonado, E. & Trigwell, S. Evaluation of tribocharged electrostatic beneficiation of lunar simulant in lunar gravity. *Journal of Aerospace Engineering* **26**, 37-42 (2012).
21 Tang, Q., Klima, M. S. & Chander, S. A laser-optical-sheet based technique for monitoring particle charge distributions. *KONA Powder and Particle Journal* **15**, 142-149 (1997).
22 Golubovskii, Y. B., Maiorov, V., Behnke, J. & Behnke, J. Influence of interaction between charged particles and dielectric surface over a homogeneous barrier discharge in nitrogen. *Journal of Physics D: Applied Physics* **35**, 751 (2002).
23 Shao, X.-J., Zhang, G.-J., Ma, Y. & Li, Y.-X. One-dimensional modeling of dielectric barrier discharge in xenon. *Plasma Science, IEEE Transactions on* **39**, 1165-1172 (2011).
24 Cserfalvi, T. & Mezei, P. Operating mechanism of the electrolyte cathode atmospheric glow discharge. *Fresenius' journal of analytical chemistry* **355**, 813-819 (1996).
